# Coarse-to-Fine Network-Based Intra Prediction in Versatile Video Coding

**DOI:** 10.3390/s23239452

**Published:** 2023-11-27

**Authors:** Dohyeon Park, Gihwa Moon, Byung Tae Oh, Jae-Gon Kim

**Affiliations:** Department of Electronics and Information Engineering, Korea Aerospace University, Goyang 10540, Republic of Korea; dhpark@kau.kr (D.P.); ghmoon@kau.kr (G.M.); byungoh@kau.ac.kr (B.T.O.)

**Keywords:** video compression, video coding, Neural Network-Based Video Coding (NNVC), intra prediction, Versatile Video Coding (VVC)

## Abstract

After the development of the Versatile Video Coding (VVC) standard, research on neural network-based video coding technologies continues as a potential approach for future video coding standards. Particularly, neural network-based intra prediction is receiving attention as a solution to mitigate the limitations of traditional intra prediction performance in intricate images with limited spatial redundancy. This study presents an intra prediction method based on coarse-to-fine networks that employ both convolutional neural networks and fully connected layers to enhance VVC intra prediction performance. The coarse networks are designed to adjust the influence on prediction performance depending on the positions and conditions of reference samples. Moreover, the fine networks generate refined prediction samples by considering continuity with adjacent reference samples and facilitate prediction through upscaling at a block size unsupported by the coarse networks. The proposed networks are integrated into the VVC test model (VTM) as an additional intra prediction mode to evaluate the coding performance. The experimental results show that our coarse-to-fine network architecture provides an average gain of 1.31% Bjøntegaard delta-rate (BD-rate) saving for the luma component compared with VTM 11.0 and an average of 0.47% BD-rate saving compared with the previous related work.

## 1. Introduction

Recently, Ultra High Definition (UHD) high-quality videos have become dominant, along with the increasing popularity of virtual reality content, 360-degree videos, and volumetric videos. These high-quality video services have led to a substantial increase in the demand for massive video data in broadcasting, mobile, and streaming services [1]. However, the memory resources and transmission capacities for such video services are still limited. In response, the Joint Video Experts Team (JVET) of the ITU-T Video Coding Experts Group (VECG) and the ISO/IEC Moving Picture Experts Group (MPEG) developed Versatile Video Coding (VVC), a new video coding standard that surpasses the compression capabilities of High Efficiency Video Coding (HEVC) [2]. Compared with HEVC, VVC provides approximately a 50% bit-rate reduction while maintaining the same perceptual visual quality. Furthermore, VVC is designed to accommodate various types of video content, including 8 K high resolution, high dynamic range, screen content, and 360-degree videos. VVC is also well suited for diverse video applications such as scalable resolution, layered coding, and flexible bitstream access [3].

In VVC, various new coding technologies are employed to enhance coding performance while preserving the block-based hybrid coding structure. Some core coding tools that enable flexible block partitioning can bring significant improvements. The Multiple Type Tree (MTT) structure, which includes binary trees, ternary trees, and quadtrees, provides flexible block partitioning, allowing for various shapes and sizes of rectangular coding blocks within the coding tree unit [4]. Geometric Partitioning Mode (GPM) enables nonrectangular partitioning by utilizing weighted blending of the bi-directional inter predictions [5]. Additionally, individual coding tools in each module, such as intra prediction [6], inter prediction [7], transform [8], quantization [9], in-loop filter [10], and entropy coding [9], offer significant advantages in terms of efficient video compression.

VVC incorporates several newly adopted coding tools that leverage machine learning techniques. One of these tools is Matrix-based Intra Prediction (MIP), a low-complexity neural network-based intra prediction. MIP utilizes matrices that are learned from extensive training datasets [11]. It predicts the current block by performing matrix-vector multiplication using a pre-trained matrix and a vector of down-sampled neighboring reference samples. The matrix is selected among several pre-defined matrices, and its index is explicitly signaled. To reduce the size of hard-coded matrices and computational complexity, the output samples of matrix-vector multiplication are up-sampled for the final prediction of MIP. Another improvement in VVC is the Low-Frequency Non-Separable Transform (LFNST), which is based on neural networks and applied to the low-frequency region of the discrete cosine transform (DCT) coefficients. LFNST achieves superior energy-compaction results [12]. The LFNST transform kernels were trained using a dataset composed of the primary transform coefficient blocks of the intra-predicted residuals. The set of LFNST kernels is implicitly determined based on the intra prediction mode, and the index indicating the applied LFNST kernel is explicitly transmitted.

As previously mentioned, machine learning-based video coding tools have been extensively investigated for their promising performance during the development of VVC. Following the standardization of VVC in July 2020, the JVET launched Exploration Experiments on Neural Network-Based Video Coding (NNVC EEs) to explore and evaluate the performance and suitability of these tools as next-generation video coding standard technologies [13]. The NNVC EEs primarily focus on coding tools in categories such as in-loop filter, post-filter, super-resolution, and inter/intra prediction, which have demonstrated significant coding gains in terms of rate distortion (RD) cost. Apart from the JVET activities, numerous deep learning-based video coding technologies have also contributed to enhancing compression performance and accelerating the VVC encoding process [14].

Intra prediction plays a crucial component of the block-based hybrid video coding scheme, where it estimates the current block based on adjacent samples from the previously reconstructed blocks. The intra prediction process can be summarized as follows: Firstly, using previously reconstructed samples from adjacent blocks of the current block, reference samples are constructed. Then, these constructed reference samples are mapped to predict samples for the current block using various directional patterns. Finally, the best pattern with the minimum RD cost among various patterns is selected, and the final prediction sample is generated using that selected one. Traditional intra prediction relies on predefined prediction modes to reduce spatial dependencies. VVC employs DC and planar modes, like HEVC. Additionally, angular intra prediction modes provide finer and wider angles with 93 directionalities, expanding on the 33 angular modes of HEVC. Moreover, VVC introduces MIP and Multiple Reference Line (MRL) predictions to generate additional prediction candidates complementing the conventional intra modes. However, these approaches may not effectively handle images containing complex spatial features or multiple directional edges.

To address the limitations, this paper proposes a neural network-based approach as an additional intra coding tool in VVC. The proposed network adopts the concept of the coarse-to-fine network architecture to generate refined prediction samples. Furthermore, the network considers the importance of reference samples at each position to enhance prediction performance. The proposed intra prediction mode is integrated into the VVC Test Model (VTM) with codec optimizations, including efficient intra mode signaling and intra mode mapping. The contributions of the proposed method are as follows:

- An intra prediction method based on Coarse-to-fine networks that employ both convolutional neural networks and fully connected layers is proposed to enhance the coding efficiency of VVC intra prediction.
- The coarse network is designed to adjust the impact on prediction performance based on the positions and conditions of reference samples.
- The fine network generates improved prediction samples by considering continuity with adjacent reference samples and facilitates prediction through upscaling at block sizes not supported by the coarse network.
- A dataset construction method, considering block partitioning and an objective function based on transform and quantization, is proposed to enhance network usability in video codecs.

Experimental results demonstrate that our Coarse-to-fine network architecture provides significant coding gain compared with VTM 11.0.

The structure of this paper is as follows: Section 2 provides a brief overview of state-of-the-art intra coding methods based on machine learning. Section 3 introduces the proposed coarse-to-fine network for intra prediction, incorporating concepts of position-wise utilization, reference sample weighting, and prediction block refinement using simple and efficient neural networks. Section 4 describes the training methods for the proposed network and outlines the necessary modifications to integrate the trained network into a video codec. Section 5 presents the experimental results comparing the proposed method with the state-of-the-art model. The statistical analysis of the proposed intra mode and visual examples is also demonstrated in Section 5. Finally, Section 6 concludes the paper.

## 2. Related Works

In VVC, intra prediction is performed with a set of predefined modes that can predict a block, characterized by a dominant directionality or smoothness. Although MIP is based on machine learning techniques, it is difficult to predict a coding block with complex textures, similar to other intra prediction modes, since the learned parameters are associated with a simple linear function. Furthermore, general approaches to predictive coding are conducted by selecting the best prediction mode in terms of RD cost. These approaches also include signaling mode information to indicate the selected mode for the decoder. Accordingly, the number of feasible intra prediction modes is restricted to maintain an acceptable trade-off between prediction performance and bit overhead for mode signaling.

A neural network-based approach is employed to address the issues associated with intra prediction because neural networks can approximate diverse intra predictive functions, especially for coding blocks with complex features. These issues can be resolved if adequate training data are provided for the network parameters. Neural network-based intra-picture compression technologies are being actively researched, focusing on tool-level coding within a legacy video codec and picture-level end-to-end coding.

Many machine learning-based algorithms have been introduced as coding tools for an intra prediction module of traditional block-based hybrid video coding architectures, such as HEVC and VVC. As discussed previously, MIP generates multiple predictions through multiple modes, using hardcoded sets of weights trained to consist of different prediction properties. Pfaff et al. [15] have reduced the MIP prediction complexities and the memory requirement to store the pre-learned weights. These simplifications facilitate the integration of MIP into the VVC standard as the first coding tool using machine learning techniques [16]. Sun et al. [17] presented the intra prediction modes that integrate the multiple networks into the HEVC based on fully connected layers. The network is methodologically similar to MIP. However, it is distinct in diverse aspects, such as layer depth, number of prediction modes, and reference sample configuration. These authors further present a probability-aware, efficient mode signaling method to enhance the coding performance of the prediction mode. Hu et al. [18] proposed a progressive spatial recurrent neural network (PS-RNN) to improve the intra prediction performance of HEVC. PS-RNN progressively generates the prediction block by fully using the spatial correlations via three recurrent units. Dumas et al. [19] proposed a set of neural network architectures consisting of fully connected and convolutional networks that delivered better prediction performance in larger blocks, which comprised neighboring reference samples with complex textures. These authors argued that the coding performance of the proposed intra prediction was typically obtained from the coding blocks with complex textures when integrated into the HEVC’s intra prediction module. Brand et al. [20] introduced the concept of conditional autoencoder (CAE)-based intra prediction for all color components. The CAE-based method can generate several prediction modes using one network. A latent representation obtained from the encoder network should be transmitted instead of the prediction mode signaling, and the latent representation is fed into the decoder network along with neighboring reference samples to generate the prediction block. Studies [17,18,19] have explored HEVC enhancement, whereas the CAE network [20] is integrated into VVC to evaluate the coding performance. Therefore, the CAE network experimented with on VVC is suitable for experimental comparison with our proposed method. Furthermore, Dumas et al. [21] employed a neural network architecture capable of predicting LFNST mode as well as intra prediction for VVC enhancement. However, this study is inappropriate for comparison with the proposed method because it includes a transform selection process as well as NN-based intra prediction. Blanch et al. [22] described the effectiveness of attention-based neural networks for chroma intra prediction. Moreover, simplifications in the network architecture are proposed to reduce the coding complexity in VVC. In addition to generating intra-prediction blocks based on neural networks, active research is being conducted on acceleration algorithms for intra prediction using neural networks. Park et al. [23] proposed a machine learning-based early skip mode decision method for VVC intra sub-partitioning, which led to an average 7.2% decrease in encoding runtime with minimal coding loss.

Image inpainting aims to restore the damaged pixel features of the incomplete image. Image inpainting is similar to the intra prediction for generating approximations of original samples in an incomplete image. Recently, various image-painting methods based on machine learning have been introduced to improve quality performance on the basis of a two-stage coarse-to-fine network architecture. The first stage makes an initial coarse prediction, and the second stage predicts the final output, taking the coarse outcome as input [24]. Thus, the fine network can learn better feature representation than the coarse network, and the concept of the coarse-to-fine network is like deep residual learning. Yu et al. [25] present simplified coarse and refinement networks by replacing all vanilla convolutions with gated convolutions, in which the size of the proposed model is reduced by 25% compared with the previous model without performance degradation. However, the network size and complexity are still too heavy to fit into a video codec. Moreover, coarse-to-fine networks are widely adopted and studied as solutions to enhance the degraded image quality resulting from various video processing outcomes. Jin et al. [26] proposed the Progressive Motion-Texture Synthesis Network (PMSN) to address challenges in existing video frame synthesis tasks related to improving perceptual quality and maintaining semantic representation capability. Additionally, Luo et al. [27] introduced a new Coarse-to-Fine Spatio-Temporal Information Fusion (CF-STIF) for enhancing the quality of loss-compressed videos.

In this paper, only the concept of coarse-to-fine architecture is used for the proposed method to maintain a reasonable level of network complexity acceptable to video codecs. This is unlike previous image-painting methods based on neural networks, which are described by an enormous number of parameters and operations. 

## 3. Proposed Coarse-to-Fine Network for Intra Prediction

**Figure 1:**
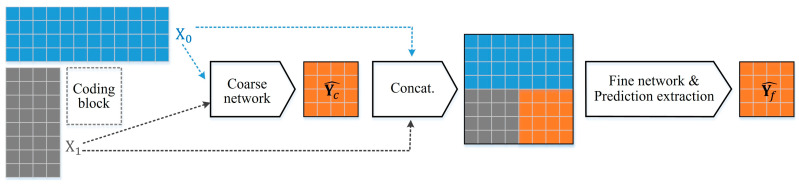
The overall process of the proposed intra prediction based on the coarse-to-fine network.

As depicted in Figure 1, the proposed network structure is designed to adopt the concept of coarse-to-fine architecture, which is widely recognized in the field of image inpainting. The two-stage structure enables diverse predictions by employing separated networks that can be trained for specific purposes. Moreover, the training process of the coarse-to-fine network follows a similar approach to residual learning, ensuring stability during training [22]. However, due to the computational complexity, the coarse-to-fine network may not be suitable for video codecs that perform recursive partitioning optimization. Consequently, previous works on NN-based intra prediction have focused on one-stage concepts [15,16,17,18,19,20,21,22]. To leverage the advantages of the coarse-to-fine network structure without introducing additional complexity, we have devised a simple network structure that solely incorporates the coarse-to-fine concept.

Specifically, the proposed coarse network generates prediction samples $\hat{Y_{c}}$ by taking advantage of its different benefits, which depend on the position and status of the neighboring reference samples. The coarse network analyzes the general directionality of a neighboring context and approximates prediction samples. The fine network, composed of simple convolutional neural networks (CNNs), conducts the quality enhancement or super-resolution of the prediction samples generated by the coarse network to produce the final prediction samples $\hat{Y_{f}}$. The fine network reproduces prediction samples closer to the original samples than coarse prediction samples, in terms of the connectivity of the neighboring context and spatial feature representation. 

We present a detailed description of the proposed coarse and fine networks for intra prediction in the next subsections.

### 3.1. Coarse Networks

Previous neural network-based models [15,16,17,18,19,20,21,22] have the shortcoming that they are difficult to reflect the positional characteristics and importance of reference samples according to image context. Previous intra prediction network architectures connected reference and prediction samples directly. Moreover, the architectures are not designed to consider the avaliability of reference samples. We propose the intra prediction network to address these issues, which is capable of responding to many situations that can occur in the actual video encoding, e.g., unavaliable reference samples. To adaptively specialize in different situations, the weighting process based on neighboring contexts is applied to reference samples before generating a prediction block. Thus, our coarse network comprises two parts: feature recalibration and prediction. In the feature recalibration, the importance of the reference samples divided into block units is determined according to the locations and characteristics of the reference samples. Then, the prediction part induces preliminary prediction samples through fully connected layers, inputting the position-wise weighted features.

**Figure 2:**
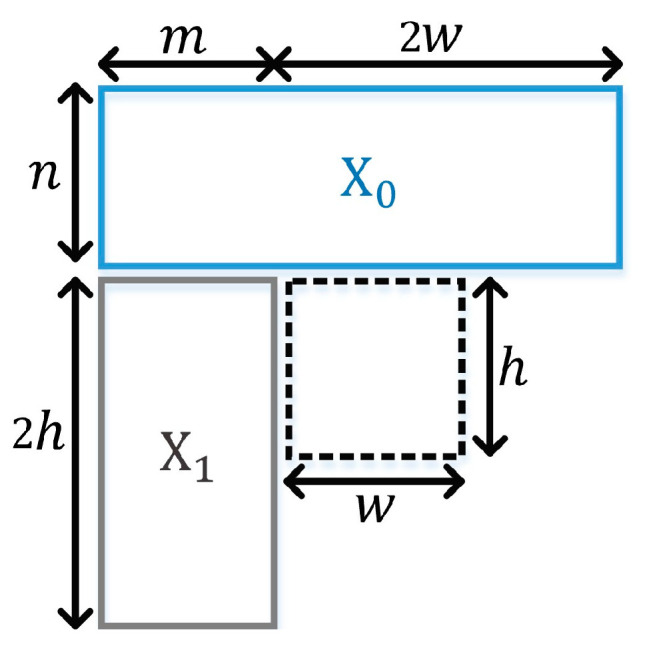
The illustration showing reference areas $X_{0}$ and $X_{1}$ of the current coding block.

Figure 2 shows neighboring reconstructed blocks $X_{0}$ and $X_{1}$, located on the above and left sides of the current coding block to be predicted. The values of m and n are set, depending on the width w and height h of the coding block. If w or h is smaller than 16, m and n is set to 8. Otherwise, m and n are set to w/2 and h/2, respectively.

**Figure 3:**
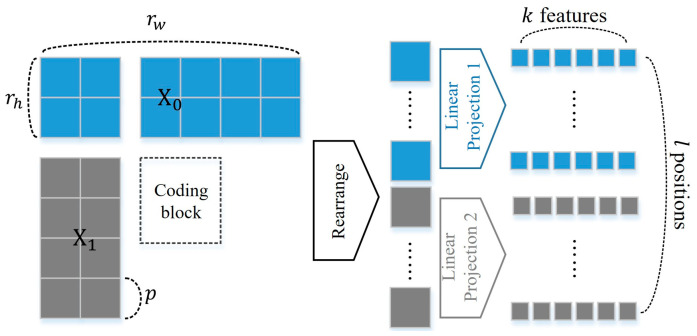
The process of the position-wise linear projection in the proposed coarse network.

As shown in Figure 3, two-dimensional reference samples $X\in R^{r_{h}\times r_{w}}$ is reshaped into a sequence of one-dimensional features $X\in R^{l\times p^{2}}$ for each side of the reconstructed block, according to two-dimensional patches, where $r_{w}$ and $r_{h}$ denote the width and height of the reference sample block, respectively. Moreover, p is the size of each square patch, and $l=r_{h}r_{w}/p^{2}$ is the number of patches. Here, the value of patch size p is set to 4, and the number of projected features k is set to 16. Then, the reshaped position-wise reference samples are mapped to k features with a trainable linear projection matrix E [28]. We refer to the output of the linear projection as the position-wise embedding matrix $X^{\prime }\in R^{l\times k}$, which is derived as follows:

(1) $$X^{\prime }=\left[X_{1}E;X_{2}E;\cdots ;X_{l}E\right],$$

where $E\in R^{p^{2}\times k}$ and vector x denotes the flattened two-dimensional patch for each position within X. The linear projection is conducted for the above and left reference blocks, and related trainable parameters are also maintained separately.

**Figure 4:**
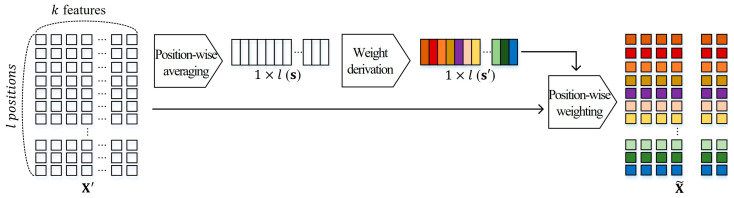
The process of the position-wise feature recalibration in the proposed coarse network.

Figure 4 shows the position-wise feature recalibration process. The feature recalibration $f_{fr}:X^{\prime }\to \tilde{X}$, $X^{\prime }\in R^{l\times k}$, $\tilde{X}\in R^{l\times k}$ is conducted to adjust the influence of the position-wise embeddings, according to the importance. This is to explicitly suppose the correlations between the positions of its patches [29]. In detail, average pooling is performed to generate scalar $s_{i}$, which is the value of i-th position in the position-wise statistic vector s, calculated by the following:

(2) $$s_{i}=\frac{1}{k}\sum_{j=0}^{k}X_{i}^{\prime }(j).$$

To obtain $s^{\prime }$ that reflects the position-wise dependencies, two fully connected layers with activation functions are defined as follows:

(3) $$s^{\prime }=g\left(W_{2}\delta \left(W_{1}s\right)\right),$$

where $W_{1}\in R^{l\times l}$, $W_{2}\in R^{l\times l}$; g and δ refer to Sigmoid [30] and ReLU [31] activation functions, respectively. The output $\tilde{X}$ of the feature recalibration is obtained by applying derived weights $s^{\prime }$ to position-wise embeddings $X^{\prime }$ as follows:

(4) $${\tilde{X}}_{i}={s_{i}^{\prime }X}_{i}^{\prime },$$

where $\tilde{X}=\left[{\tilde{X}}_{1};X_{2};\cdots ;X_{l}\right]$ is the position-wisely weighted embeddings.

The coarse prediction samples are obtained based on the prediction part defined in Table 1. The predictive part in the coarse network is composed of four fully connected layers with $W_{3}\in R^{lk\times r}$, $\{W_{4}$, $W_{5}\}\in R^{r\times r}$, and $W_{6}\in R^{r\times wh},$ where r is the number of neurons in the prediction part and is set to 1024. Then, the prediction samples are reshaped with a raster scan to form the shape of the coding block. Except for the last layer, each fully connected layer is followed by a LeakyReLU [32] non-linear function σ with a slope of 0.1. The architecture of the coarse networks is described in Table 1. The parameters of the proposed coarse network are trained and maintained differently for luma coding blocks with eight different sizes in {4 × 4, 4 × 8, 8 × 8, 4 × 16, 8 × 16, 16 × 16, 4 × 32, 32 × 32}.

### 3.2. Fine Networks

**Figure 5:**
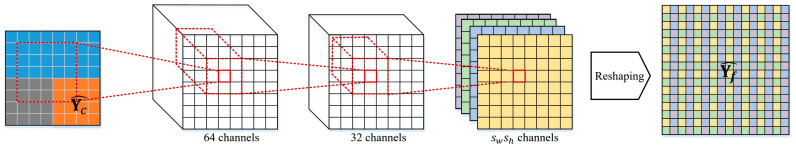
The architecture of the proposed fine network for refinement and upscaling.

In fully connected layer-based intra prediction, the correlation between prediction samples and connectivity between reference lines and a prediction block may deteriorate due to the individual generation of prediction samples based on their positions within the coding block. To address these issues and enhance the prediction quality, we introduced a fine network that includes refinement networks and up-scaling networks. These networks are based on uncomplicated CNNs, as illustrated in Figure 5. The network structures for refinement and up-scaling are the same, except for the number of output channels of the last convolutional layer, according to the scale factors pre-determined by the block size. The fine network is composed of three convolutional neural networks with the hyperbolic tangent function for nonlinearity. $W_{7}$, $W_{8}$, and $W_{9}$ denotes learnable convolutional parameters, as presented in Table 2.

As shown in Figure 5, the coarse prediction block is concatenated with the above and left reference blocks and used as input for the fine networks. The refinement network and the up-scaling network use the same structure, except that the number of output channels of the last layer is determined according to the scale factors $s_{h}$ and $s_{w}$ for the height and width of the coding block. If at least one of the scale factors is greater than 1, the coarse network defined for a smaller block is applied by conducting average pooling with a filter size of ($s_{h}$, $s_{w}$) for the reference samples. Then, the output of the up-scaling network is rearranged to form the final prediction block of shape h×w. For example, average pooling is performed on the reference samples for a 64 × 64 block, and the reduced reference samples are fed into the coarse network for a 32 × 32 block. Then, the output of the coarse network is used as the input of the fine network for the current coding block of 64 × 64. The fine prediction consists of 11 neural networks for all possible block sizes in VVC.

## 4. Training Considering Video Codec

### 4.1. Dataset Construction

A training dataset is acquired based on the block structure obtained in the actual video encoding results to reflect the block partitioning determined in terms of RD cost. The network training is conducted using an individual dataset configured according to the shape and size of a coding block. We use a video database consisting of BVI-DVC [33] and TVD [34] to construct the training set. In particular, BVI-DVC can achieve significant improvement in video coding performance, which contains 800 progressive scanned video sequences in four spatial resolutions of a range of 270 p–2160 p along with miscellaneous representative contents.

All sequences in the database are encoded with VTM 11.0 at five quantization parameters (QPs) of 22, 27, 32, 37, and 42 [35]. Then, the training dataset is built with pairs of an original coding block and its reconstructed reference block based on the block partition results of real encoding. In more detail, one frame per eight frames is selected to reduce the temporal redundancy of video sequences. At most, 40 data pairs are extracted per frame to avoid biased representation in high-resolution frames. Ultimately, the training set is composed of approximately 2.5 M pairs for each block size.

**Figure 6:**
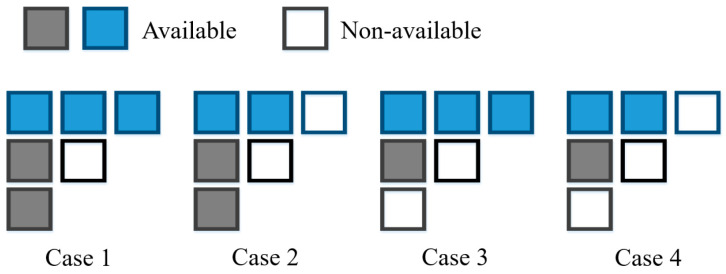
Cases of availabilities of neighboring reference parts.

Generally, the unavailable reference samples are copied clockwise from the closest available samples. However, this copy process is not used for the unavailable reference samples in the proposed method since simple padding in multiple reference lines may fabricate an unnecessary directionality within the reference block, which can confuse the training process of the intra prediction network. Thus, we fill the unavailable reference samples with the mean value of the available samples instead of copying them. Moreover, the proposed networks are trained and adjusted to different cases of the availabilities of neighboring blocks, as shown in Figure 6. In other words, the training data corresponding to each case occupies the entire training set in proportion to the analyzed probabilities based on the experimental observation.

The proposed networks are trained for the luma component, considering that the luma component contains most of the visual information. Thus, the networks trained for the luma component are applied to the intra prediction of chroma components.

### 4.2. Objective Function

Generally, the cost associated with the mean square error (MSE) is taken as an objective function for minimizing the difference between the ground truth block and its estimation. However, the MSE is an unsuitable metric for training networks that will be integrated into video codecs. This is different from measuring optimized compression performance in video codecs such as HEVC and VVC. DCT, which efficiently compacts the energy of image signals, is also used to transform the residual signal in video codecs.

Thus, we propose a weighted DCT-based objective function to learn the parameters of the coarse-to-fine networks. The weight matrix Q is applied to the transform coefficients in the unit of 4 × 4 coefficient group (CG) to reflect the adaptive quantization, according to the positional importance of the transform coefficients [36]. The exponential weight factor is employed since the distribution of DCT coefficients follows the Laplacian distribution [37]. According to the diagonal scan order [9], the weight $q_{i}$ for the i-th position in the weight matrix Q is derived as follows:

(5) $$q_{i}=e^{-\left(\frac{i}{b}\right)},$$

where b denotes the number of CGs in the coding block. Then, the size of Q is upscaled by four times for each row and column so that the same weight value of Q is applied to each transform coefficient in the unit of CG.

Both coarse and refinement networks are simultaneously trained by minimizing the weighted DCT-based objective function defined as follows:

(6) $$\underset{\theta_{c},\;\theta_{r}}{\min}{\left\|\left(H\cdot D\cdot H^{T}\right)\circ Q\right\|}_{1},\;D=Y-f_{r}\left(f_{c}\left(X;\theta_{c}\right);\theta_{r}\right),$$

where H represents a one-dimensional DCT-II matrix and · and ∘ operators are matrix multiplication and element-wise multiplication, respectively. Moreover, $\theta_{c}$ and $\theta_{r}$ denote the parameters of the coarse network and refinement network, respectively. Similarly, up-scaling networks are trained as follows:

(7) $$\begin{array}{c} \underset{\theta_{u}}{\min}{\left\|\left(H\cdot D\cdot H^{T}\right)\circ Q\right\|}_{1},\\ D=Y-P\left(f_{u}(f_{c}\left(X^{red};\theta_{c}\right);\theta_{u})\right), \end{array}$$

where $\theta_{u}$ is the parameters of the up-scaling network and P is a reshaping operator to align the output vector of ($s_{h}s_{w}\times h/s_{h}\times w/s_{w}$) to the prediction block of (h ×w). $X^{red}$ denotes the downscaled input vector according to the average pooling with the $s_{h}$ and $s_{w}$ parameters defined in Table 3. During this training, the coarse network for the reduced block size is frozen. In other words, only the up-scaling network is trained, whereas the coarse network just serves the prediction block of reduced block size.

The size of the minibatch is set to 64, and the number of iterations is more than 40 K for one epoch. We trained the proposed networks using the ADAM [38] optimizer during 100 epochs. We applied the learning rate reduction method by multiplying 0.77 by the previous rate for every 10 epochs, based on the initial learning rate and minimum learning rate settings of 0.0005 and 0.00001, respectively. 

## 5. Codec Integration

### 5.1. Network Integration in VVC

In the configuration of VVC intra coding, the size of coding units (CUs) varies from 4 × 4 to 64 × 64, following the MTT block structure, which allows all rectangular shapes. The proposed coarse and refinement networks are embedded in VTM for eight block sizes of {4 × 4, 4 × 8, 8 × 8, 4 × 16, 8 × 16, 16 × 16, 4 × 32, 32 × 32}. Additionally, the up-scaling networks are embedded into 8 × 32, 16 × 32, and 64 × 64 blocks. According to the block size, the coarse network and one of the refinement and up-scaling networks are sequentially operated with transposition and down-sampling of reference samples to generate the final prediction block, as shown in Table 3.

To evaluate the proposed method in terms of coding efficiency, the coarse-to-fine neural network-based intra prediction module is integrated into VTM as an additional specific intra prediction mode instead of entirely substituting the intra prediction module of VVC. A rate distortion optimization (RDO) process is conducted to decide the best mode among the neural network-based intra mode and VVC’s intra prediction modes on the encoder side. For this reason, an additional flag is transmitted to indicate whether the proposed mode is selected at the CU level. Furthermore, the planar mode flag adopted in the VVC as an efficient intra mode coding because of its highest selectivity is removed since the proposed additional intra mode changes the selectivity distributions of all available modes, especially the planar mode.

### 5.2. Mode Mapping for LFNST

In the VVC, the LFNST is applied to the transform coefficients of DCT-II to exploit the remaining correlations of the transformed coefficient. The LFNST kernels are defined as four sets, comprising two kernels each. The set to be selected is predefined according to the conventional intra mode. Thus, a mapping process that converts a neural network-based mode to a regular intra mode is required for applying LFNST to the blocks coded with a coarse-to-fine intra prediction network.

Due to the network-based intra prediction that generates diverse textures, we devised a mapping method to convert the proposed network-based intra mode into an appropriate regular mode according to the prediction block characteristics within the LFNST procedure. A histogram of regular intra modes is derived based on the methodology of Decoder-Side Intra Mode Derivation (DIMD) to select a regular intra mode that fits the current prediction block [39]. Gradients $g_{h}$ and $g_{v}$ are calculated by applying the horizontal and vertical Sobel filters for each prediction sample obtained from the neural network-based mode to find a regular prediction mode suitable for the current prediction block. Then, if both gradients are greater than the threshold t, the amplitude of the gradients is accumulated in the histogram as follows:

(8) $$Hist\left[im\right]=Hist\left[im\right]+\left|g_{h}\right|+\left|g_{v}\right|,$$

where im denotes an index of derived regular intra mode by conducting the angle-to-mode mapping in DIMD. Otherwise, the histogram is updated by accumulating the inverse amplitude in the histogram for planar mode (pl) as follows:

(9) $$Hist\left[pl\right]=Hist\left[pl\right]+2t-\left|g_{h}\right|-\left|g_{v}\right|.$$

Finally, a single regular intra mode is selected, which corresponds to the tallest spike of the histogram and is considered an alternative to the neural network-based intra mode in LFNST.

## 6. Performance Evaluation

### 6.1. Evaluation of Coarse-to-Fine Network

We progressively compare the coding performances of intra prediction networks with key features to evaluate the proposed coarse-to-fine network structure. First, *Coarse*−, which is a simplified version of the coarse network by removing the position-wise reference feature scaling parts defined in (1) to (3), is evaluated. That is, *Coarse*− is compared with *Coarse*, which is the coarse network only, to evaluate the effectiveness of feature scaling for each reference position. Note that, the processing in (1) to (3) only introduces an average 7% parameter increase. Subsequently, an evaluation of the entire coarse-to-fine network (*Coarse-to-fine*) is conducted. These three networks with different structures are individually trained through the training method described in Section 4. Currently, JVET is developing a version of VTM called NNVC, which is specifically designed for the validation and assessment of various neural network-based tools [40]. However, to evaluate the performance of the proposed method, we integrated the three networks into VTM 11.0, which is used as the basis for NNVC, as an additional intra prediction mode. Under JVET common test conditions, an all intra (AI) configuration of VVC is used for the video coding experiments [41], and the four rate points are specified with corresponding QP values of 22, 27, 32, and 37. The test videos include all sequences of classes A, B, C, D, and E with varying resolutions from 400 p to 4 Kp. The VTM 11.0 results are the anchor used for experimental comparison, and the encoder and decoder with the four QPs share the same trained neural network models.

The compression performances of the *Coarse*−, *Coarse*, and *Coarse-to-fine* networks with respect to VVC are provided in Table 4. The Bjøntegaard delta-rate (BD-rate) [42], which is the method of finding numerical averages between RD curves, is used to measure the coding performance of test sequences for each color component. The coding performance of the coarse network with the position-wise reference feature scaling is better than that of the simplified coarse network, *Coarse*−. The position-wise reference feature scaling method offers an average of 0.18% BD-rate gain in peak signal-to-noise ratio for the luminance component (Y-PSNR). Furthermore, the fine network yields an additional 0.04% BD-rate gain of Y-PSNR. Overall, a significant bit saving of 1.26% on average is observed for the BD-rate of Y-PSNR compared with the anchor VTM 11.0.

### 6.2. Visualization Analysis

**Figure 7:**
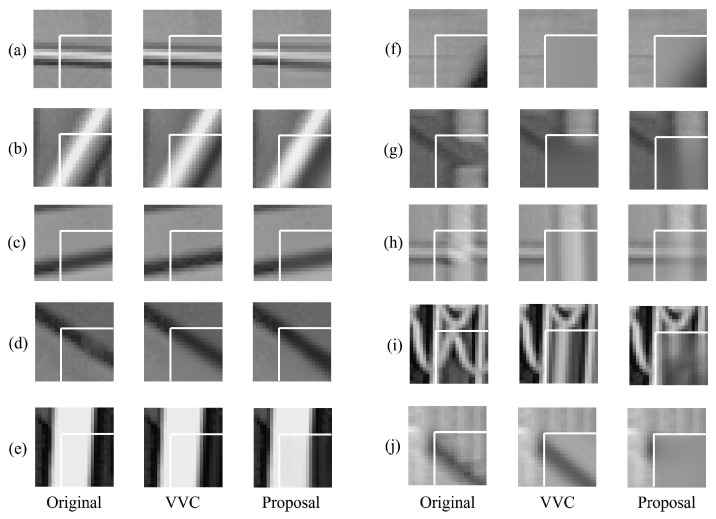
Examples of prediction blocks and their spatial contexts (**Left**: original blocks to be predicted; **Middle**: prediction blocks by the best intra mode in VTM 11.0, labeled VVC; **Right**: prediction blocks of Coarse-to-fine network, labeled Proposal). The white lines denote boundaries between prediction blocks and their spatial contexts.

We visualized the prediction signals of our course-to-fine networks, ground truth signals, and VVC’s predictions to analyze the subjective quality improvement. Figure 7 compares the prediction blocks of the proposed networks and the best VVC mode for the coding blocks of 16 × 16. The best VVC mode is an intra prediction mode selected as the best mode based on RDO in AI modes, including MIP in the VTM 11.0 encoder. Overall, as shown in Figure 7, the coarse-to-fine network makes predictions more flexible depending on the characteristics of the input reference samples. Some examples of this property are demonstrated as follows: For the angular prediction cases shown in Figure 7a–e, the coarse-to-fine network can cover traditional angular modes without signaling, which is required to indicate the multiple predefined modes. Figure 7f,g show that the proposed network generates valid predictions even when the prediction performance of VVC modes can be limited due to the distance from reference samples or occlusion in the dominant direction. Moreover, as shown in Figure 7h,i, it is verified that sufficiently meaningful predictions can be generated in a block in which two directions exist simultaneously or in a block with a more complex texture. However, Figure 7j shows that the network-based intra prediction mode has some limitations in the prediction compared with a conventional method when the reference samples do not have sufficient context for the prediction. Because only a single prediction is available in the network-based prediction mode, whereas the conventional method selects the best predictor based on the RDO process among multiple candidates.

**Figure 8:**
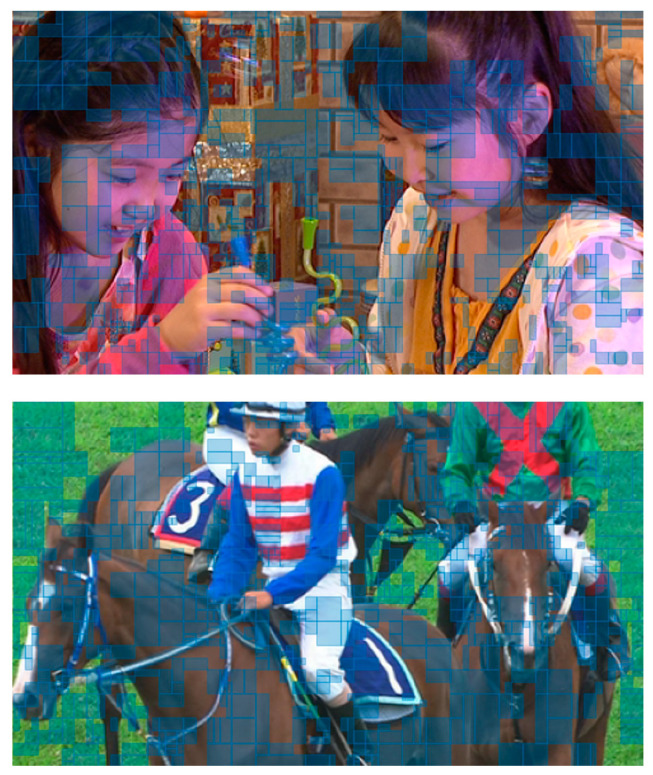
Examples of coded blocks as the coarse-to-fine network-based mode based on RDO (blue-shaded blocks) (Above: BlowingBubbles, Below: RaceHorses, QP: 37).

As shown in Figure 8, we visualized the coding blocks that selected the proposed network-based mode as the best mode in the encoding experiment. For QP 22, 27, 32, and 37, approximately 35%, 40%, 45%, and 50% areas of the entire frame are coded by the coarse-to-fine network mode, respectively. Additionally, the proposed mode is selected at rates of approximately 27%, 30%, 35%, and 40% for QP 22, 27, 32, and 37, respectively. These tendencies in the area and selection ratio of the proposed mode by QP reflect the coding gain according to QP. That is, the coding gain tends to increase as the QP increases. This is because the RD costs of the conventional prediction modes increase in the high QP range due to the mode signaling.

### 6.3. Complexity Analysis

As shown in Table 5, the proposed coarse-to-fine network has a huge computational complexity, which is 6–9 times the encoding runtime of VVC and about 80 times the decoding runtime of VVC. Table 5 also shows an increase in encoding runtime when *Coarse*−, *Coarse* and *Coarse-to-fine* network-based intra prediction modes are integrated into VTM 11.0. Directly comparing the decoding runtimes with existing methods is difficult due to variations in experimental environments and the use of NN libraires. However, the proposed method is superior to other NN-based intra prediction methods reported to have about 200 times the decoding runtime of VVC [18]. Nevertheless, the decoding complexity is still high, and further studies are needed to reduce the complexity of NN operations. The experiments were conducted on an Intel Xeon Gold 6256 3.6 GHz processor with a GCC 9.4.0 compiler under an Ubuntu 20.04.4 LTS. The networks, integrated into the VTM, are processed on a CPU basis, and only a single thread is used in the experiments.

As a result, the runtime of the *Coarse* network increases by approximately 14% compared with the *Coarse*− network with the additional operation of position-wise scaling, and the runtime of the *Coarse-to-fine* network increases by about 32% compared with the *Coarse* network.

### 6.4. Conparison with State-of-the-Arts

The proposed coarse-to-fine network is compared with the CAE-based network [20], which is a neural network-based intra prediction for VVC. The CAE networks support the intra prediction modes for variable block sizes, suitable for VVC’s MTT. The proposed networks were implemented in VTM 6.2 and experimented under AI configurations with MIP turned off to maintain the same experimental setups as described in [20].

As shown in Table 6, the coarse-to-fine network offers a better prediction with significant BD-rate gains of 1.01% and 0.47% than the VVC’s MIP and CAE networks.

## 7. Conclusions

This paper introduces a coarse-to-fine network architecture aimed at enhancing the intra prediction performance of VVC. The coarse network is designed to take into account the characteristics of reference samples based on their positions and spatial context, utilizing feature recalibration to adjust feature scales for improved representation of essential features in coarse predictions. The fine network enhances prediction accuracy and connectivity with adjacent samples. In addition, we propose a dataset construction method, considering block partitioning and an objective function based on transform and quantization, to enhance network usability in video codecs. Our networks, individually trained for variable block sizes, efficiently support block-based hybrid video codecs like VVC. The proposed networks are integrated into VTM as an additional intra mode. The experimental results demonstrate significant coding gains, surpassing state-of-the-art models in BD-rate savings. The method effectively addresses conventional intra prediction limitations, showcasing potential as a core intra coding tool beyond VVC. However, further enhancements in network structure, training methods, and efforts to reduce computational and memory complexity are essential, especially in scenarios with limited neighboring context.

## Figures and Tables

**Table 1:** Hyper-parameters of fully connected layer stacks for position-wise weight derivation and prediction in the proposed coarse networks.

(a) Position-Wise Weight Derivation			
Layer	Layer Type	Number of Neurons	Non-Linearity
$W_{1}$	Fully connected	l	Sigmoid
$W_{2}$	Fully connected	l	ReLU
**(b) Prediction Part**			
**Layer**	**Layer Type**	**Number of Neurons**	**Non-Linearity**
$W_{3}$	Fully connected	1024	LeakyReLU
$W_{4}$	Fully connected	1024	LeakyReLU
$W_{5}$	Fully connected	1024	LeakyReLU
$W_{6}$	Fully connected	wh	-

**Table 2:** Hyper-parameters of convolutional layer stacks for the proposed fine networks.

Layer	Layer type	Filter Size	Number of Channels	Non-Linearity
$W_{7}$	convolutional	5 × 5	64	Hyperbolic Tangent
$W_{8}$	convolutional	3 × 3	32	Hyperbolic Tangent
$W_{9}$	convolutional	3 × 3	$s_{w}s_{h}$	-

**Table 3:** Decision of transposing the reference blocks and prediction block, scaling factors for width and height, and the coarse and fine networks used for prediction for each block size available at VVC.

**(** h **,** w **)**	**(** $s_{h}$ **)**	**(** $s_{w}$ **)**	**Transposition**	Coarse Network	Fine Network
(4, 4)	1	1	no	$f_{c}^{\left(4,4\right)}$	$f_{r}^{\left(4,4\right)}$
(4, 8)	1	1	no	$f_{c}^{\left(4,8\right)}$	$f_{r}^{\left(4,8\right)}$
(8, 4)	1	1	yes	$f_{c}^{\left(4,8\right)}$	$f_{r}^{\left(4,8\right)}$
(8, 8)	1	1	no	$f_{c}^{\left(8,8\right)}$	$f_{r}^{\left(8,8\right)}$
(4, 16)	1	1	no	$f_{c}^{\left(4,16\right)}$	$f_{r}^{\left(4,16\right)}$
(16, 4)	1	1	yes	$f_{c}^{\left(4,16\right)}$	$f_{r}^{\left(4,16\right)}$
(8, 16)	1	1	no	$f_{c}^{\left(8,16\right)}$	$f_{r}^{\left(8,16\right)}$
(16, 8)	1	1	yes	$f_{c}^{\left(8,16\right)}$	$f_{r}^{\left(8,16\right)}$
(16, 16)	1	1	no	$f_{c}^{\left(16,16\right)}$	$f_{r}^{\left(16,16\right)}$
(4, 32)	1	1	no	$f_{c}^{\left(4,32\right)}$	$f_{r}^{\left(4,32\right)}$
(32, 4)	1	1	yes	$f_{c}^{\left(4,32\right)}$	$f_{r}^{\left(4,32\right)}$
(8, 32)	1	2	no	$f_{c}^{\left(8,16\right)}$	$f_{u}^{\left(8,32\right)}$
(32, 8)	2	1	yes	$f_{c}^{\left(8,16\right)}$	$f_{u}^{\left(8,32\right)}$
(16, 32)	1	2	no	$f_{c}^{\left(16,16\right)}$	$f_{u}^{\left(16,32\right)}$
(32, 16)	2	1	yes	$f_{c}^{\left(16,16\right)}$	$f_{u}^{\left(16,32\right)}$
(32, 32)	1	1	no	$f_{c}^{\left(32,32\right)}$	$f_{r}^{\left(32,32\right)}$
(64, 64)	2	2	no	$f_{c}^{\left(32,32\right)}$	$f_{u}^{\left(64,64\right)}$

**Table 4:** Experimental results comparing the BD-rate of the *Coarse*−, *Coarse*, and *Coarse-to-fine* networks with respect to VVC. The performance is evaluated using the All Intra configuration. The reference is VTM 11.0 with MIP turned off.

Video Sequence		Coarse−			*Coarse*			*Coarse-to-Fine*		
		Y	U	V	Y	U	V	Y	U	V
Class A1 (4 K)	Tango2	−1.51%	−1.76%	−1.47%	−1.76%	−1.69%	−1.88%	−1.83%	−1.26%	−1.87%
	FoodMarket4	−1.60%	−0.82%	−1.21%	−2.12%	−1.38%	−1.47%	−2.20%	−1.36%	−1.81%
	Campfire	−1.07%	−0.98%	−1.35%	−1.10%	−1.03%	−1.40%	−1.13%	−1.05%	−1.45%
Class A2 (4 K)	CatRobot1	−0.97%	−1.21%	−1.13%	−1.18%	−1.14%	−1.38%	−1.23%	−1.26%	−1.15%
	DaylightRoad2	−0.73%	−1.14%	−1.21%	−0.83%	−1.61%	−1.14%	−0.90%	−1.42%	−1.29%
	ParkRunning3	−1.05%	−0.96%	−0.95%	−1.09%	−1.01%	−1.02%	−1.04%	−1.01%	−1.02%
Class B (1080 p)	MarketPlace	−1.17%	−1.11%	−0.77%	−1.33%	−0.87%	−0.77%	−1.36%	−1.53%	−1.35%
	RitualDance	−1.59%	−0.93%	−1.13%	−1.82%	−1.21%	−1.25%	−2.00%	−1.28%	−1.46%
	Cactus	−0.99%	−1.10%	−0.96%	−1.13%	−1.08%	−0.99%	−1.12%	−1.06%	−0.85%
	BasketballDrive	−0.85%	−0.87%	−0.59%	−1.00%	−1.02%	−0.86%	−1.06%	−0.58%	−0.64%
	BQTerrace	−0.89%	−0.96%	−1.17%	−0.94%	−1.07%	−1.24%	−0.88%	−1.15%	−1.19%
Class C (WVGA)	BasketballDrill	−1.16%	−0.98%	−0.73%	−1.31%	−1.15%	−0.56%	−1.31%	−0.79%	−0.85%
	BQMall	−0.82%	−0.79%	−0.59%	−0.93%	−0.56%	−0.34%	−0.93%	−0.75%	−0.93%
	PartyScene	−0.77%	−0.51%	−0.72%	−0.79%	−0.53%	−0.67%	−0.75%	−0.45%	−0.20%
	RaceHorses	−0.90%	−1.39%	−1.14%	−1.06%	−0.85%	−1.06%	−1.10%	−0.96%	−1.12%
Class D (WQVGA)	BasketballPass	−0.71%	−0.33%	−0.95%	−0.99%	−0.35%	−0.98%	−0.88%	−0.32%	−1.03%
	BQSquare	−0.38%	−0.10%	0.12%	−0.42%	−0.32%	−0.38%	−0.41%	0.06%	0.68%
	BlowingBubbles	−1.18%	−0.25%	−1.36%	−1.26%	−0.90%	−1.43%	−1.23%	−1.32%	−1.60%
	RaceHorses	−1.06%	−1.78%	−1.23%	−1.42%	−0.27%	−1.35%	−1.46%	−0.59%	−1.60%
Class E (720 p)	FourPeople	−1.35%	−1.29%	−1.26%	−1.66%	−1.37%	−1.41%	−1.73%	−1.34%	−1.62%
	Johnny	−1.17%	−0.26%	−1.50%	−1.43%	−1.16%	−2.20%	−1.64%	−1.42%	−1.38%
	KristenAndSara	−1.02%	−0.82%	−1.00%	−1.32%	−1.08%	−0.64%	−1.43%	−1.47%	−0.36%
**Overall**	Class A1	−1.39%	−1.19%	−1.34%	−1.66%	−1.37%	−1.58%	−1.72%	−1.22%	−1.71%
	Class A2	−0.92%	−1.11%	−1.09%	−1.03%	−1.25%	−1.18%	−1.06%	−1.23%	−1.16%
	Class B	−1.10%	−0.99%	−0.92%	−1.24%	−1.05%	−1.02%	−1.29%	−1.12%	−1.10%
	Class C	−0.91%	−0.92%	−0.80%	−1.02%	−0.77%	−0.66%	−1.03%	−0.74%	−0.77%
	Class D	−0.83%	−0.62%	−0.86%	−1.02%	−0.46%	−1.03%	−0.99%	−0.54%	−0.89%
	Class E	−1.18%	−0.79%	−1.25%	−1.47%	−1.20%	−1.41%	−1.60%	−1.41%	−1.12%
**Average**		**−1.04%**	**−0.93%**	**−1.01%**	**−1.22%**	**−0.98%**	**−1.11%**	**−1.26%**	**−1.01%**	**−1.10%**

**Table 5:** Encoding and decoding runtime (RT) of the network-integrated codec compared with VTM 11.0.

Class	Enc. RT			Dec. RT
	Coarse−	*Coarse*	*Coarse-to-Fine*	*Coarse-to-Fine*
A1	508%	676%	939%	9402%
A2	603%	679%	898%	7188%
B	653%	726%	933%	7816%
C	587%	647%	850%	8549%
D	547%	599%	782%	7538%
E	684%	739%	966%	9090%
Average	594%	678%	896%	8409%

**Table 6:** Experimental results comparing the BD-rate of the proposed coarse-to-fine network with *MIP* [11] and *CAE* [20]. The performance is evaluated using the All Intra configuration. The reference is VTM 6.2 with MIP turned off.

Video Sequence		*MIP* [11]			*CAE* [21]			*Coarse-to-Fine*		
		Y	U	V	Y	U	V	Y	U	V
Class A1 (4 K)	Tango2	−1.08%	−0.44%	−0.51%	−2.20%	−3.13%	−1.92%	−2.91%	−1.51%	−2.89%
	FoodMarket4	−1.62%	−0.78%	−0.83%	−3.62%	−2.84%	−2.70%	−3.21%	−2.63%	−3.00%
	Campfire	−0.56%	−0.20%	−0.11%	−0.82%	−0.33%	−0.26%	−1.46%	−1.29%	−1.58%
Class A2 (4 K)	CatRobot1	−0.57%	−0.08%	−0.20%	−1.20%	−1.65%	−1.24%	−1.64%	−1.66%	−1.15%
	DaylightRoad2	−0.37%	0.03%	0.00%	−0.80%	−1.63%	−0.84%	−1.32%	−1.86%	−1.13%
	ParkRunning3	−0.50%	−0.18%	−0.17%	−1.05%	−0.94%	−1.01%	−1.37%	−1.08%	−1.12%
Class B (1080 p)	MarketPlace	−0.67%	−0.30%	−0.47%	−1.22%	−0.93%	−1.37%	−1.86%	−0.96%	−1.49%
	RitualDance	−0.42%	0.20%	0.16%	−1.21%	−0.92%	−1.15%	−1.95%	−0.91%	−0.65%
	Cactus	−0.48%	−0.02%	0.09%	−0.96%	−1.06%	−0.98%	−1.61%	−0.32%	−1.25%
	BasketballDrive	−0.39%	−0.52%	0.15%	−0.91%	−1.16%	−1.08%	−1.35%	0.03%	−0.03%
	BQTerrace	−0.29%	−0.03%	0.26%	−0.70%	−0.50%	−0.49%	−1.00%	−1.99%	−1.44%
Class C (WVGA)	BasketballDrill	−0.42%	−0.03%	0.17%	−0.78%	−0.54%	−0.84%	−1.85%	−0.86%	−0.09%
	BQMall	−0.50%	−0.27%	−0.50%	−0.79%	−0.96%	−1.00%	−0.76%	−0.49%	−0.88%
	PartyScene	−0.57%	0.02%	−0.27%	−0.62%	−0.56%	−0.51%	−1.05%	−1.38%	−1.25%
	RaceHorses	−0.44%	0.08%	−0.33%	−0.98%	−0.95%	−0.64%	−1.41%	−0.90%	−0.59%
Class D (WQVGA)	BasketballPass	−0.29%	−0.55%	−0.51%	−0.37%	−1.04%	−2.06%	−1.04%	3.69%	1.43%
	BQSquare	−0.74%	−0.22%	0.22%	−0.52%	−0.75%	−0.16%	−0.52%	−0.79%	−1.03%
	BlowingBubbles	−0.48%	−0.28%	−0.68%	−0.71%	−1.03%	−1.14%	−0.88%	−2.27%	−0.81%
	RaceHorses	−0.48%	−0.12%	0.20%	−0.98%	−1.21%	−0.85%	−1.79%	−1.92%	−2.75%
Class E (720 p)	FourPeople	−0.43%	−0.10%	−0.18%	−1.15%	−1.16%	−1.46%	−2.08%	−0.79%	−2.13%
	Johnny	−0.53%	−0.68%	−0.95%	−1.45%	−1.56%	−1.75%	−2.11%	−2.02%	−2.89%
	KristenAndSara	−0.46%	−0.84%	0.59%	−1.07%	−1.65%	−1.12%	−1.46%	−0.94%	0.23%
**Overall**	Class A1	−1.09%	−0.47%	−0.48%	−2.22%	−2.10%	−1.63%	−2.53%	−1.81%	−2.49%
	Class A2	−0.48%	−0.08%	−0.12%	−1.02%	−1.41%	−1.03%	−1.44%	−1.53%	−1.13%
	Class B	−0.45%	−0.13%	0.04%	−1.00%	−0.91%	−1.01%	−1.55%	−0.83%	−0.97%
	Class C	−0.48%	−0.05%	−0.23%	−0.77%	−0.75%	−0.75%	−1.27%	−0.91%	−0.71%
	Class D	−0.50%	−0.29%	−0.19%	−0.65%	−1.01%	−1.05%	−1.06%	−0.32%	−0.79%
	Class E	−0.47%	−0.54%	−0.18%	−1.22%	−1.46%	−1.44%	−1.88%	−1.25%	−1.60%
**Average**		**−0.56%**	**−0.24%**	**−0.18%**	**−1.10%**	**−1.20%**	**−1.12%**	**−1.57%**	**−1.04%**	**−1.20%**

## Data Availability

Data available in a publicly accessible repository.
